# Combinations of single doses and fractionated treatments of cis-dichlorodiammineplatinum (II) and irradiation: effect on mouse lip mucosa.

**DOI:** 10.1038/bjc.1986.212

**Published:** 1986-10

**Authors:** W. Landuyt, K. K. Ang, E. van der Schueren

## Abstract

Tolerance of the lip mucosa of NMRI mice to single and fractionated irradiation combined with cis-diamminedichloroplatinum (II) (cis-DDP) was investigated. For the various combination schedules total drug doses varying from 6 mg kg-1 to 13 mg kg-1 were injected i.p. It was found that cis-DDP did not alter the radiation sensitivity of this tissue at any of the time intervals tested (ranging from 24 h before to 72 h after single dose irradiations). When 5 daily drug injections were given concomitantly with 5 daily radiation treatments, a slight reduction of the lip mucosal reactions occurred, possibly due to partial synchronisation during treatment. No effect was seen when a single injection of cis-DDP preceded two irradiations given with increasing intervals up to 4 h. Both these combined fractionated treatment data suggest no inhibitory effect on repair of sublethal radiation damage. When repeated daily injections of cis-DDP were given in between 2 radiation doses separated by 10 days, no interference with repopulation could be detected. The present study also demonstrated an increase in systemic drug toxicity when cis-DDP was combined with irradiation, compared with that seen with either agent alone.


*Br. J. Cancer* (1986), **54**, 579–586

### Combinations of single doses and fractionated treatments of *cis*-dichlorodiammineplatinum (II) and irradiation: Effect on mouse lip mucosa

W. Landuyt, K.K. Ang* & E. van der Schueren

*Department of Experimental Radiotherapy, University Hospital St.-Rafaël, K.U. Leuven, Belgium.*

**Summary** Tolerance of the lip mucosa of NMRI mice to single and fractionated irradiation combined with *cis*-diamminedichloroplatinum (II) (*cis*-DDP) was investigated. For the various combination schedules total drug doses varying from $6\,mg\,kg^{-1}$ to $13\,mg\,kg^{-1}$ were injected i.p. It was found that *cis*-DDP did not alter the radiation sensitivity of this tissue at any of the time intervals tested (ranging from 24 h before to 72 h after single dose irradiations).

When 5 daily drug injections were given concomitantly with 5 daily radiation treatments, a slight reduction of the lip mucosal reactions occurred, possibly due to partial synchronisation during treatment. No effect was seen when a single injection of *cis*-DDP preceded two irradiations given with increasing intervals up to 4 h. Both these combined fractionated treatment data suggest no inhibitory effect on repair of sublethal radiation damage.

When repeated daily injections of *cis*-DDP were given in between 2 radiation doses separated by 10 days, no interference with repopulation could be detected.

The present study also demonstrated an increase in systemic drug toxicity when *cis*-DDP was combined with irradiation, compared with that seen with either agent alone.

*Cis*-dichlorodiammineplatinum (II), (*cis*-DDP), is an antineoplastic agent used either alone and more recently in combination with other drugs for the treatment of a variety of malignancies, including testicular, ovarian, lung, bladder and head and neck tumours. *In vitro* studies with both normal and tumour cell lines suggested that *cis*-DDP acts as a modifier of radiosensitivity (Richmond *et al.*, 1977; Douple & Richmond, 1980; Murthy *et al.*, 1979; Dritschilo *et al.*, 1979). Several studies with animal tumours also revealed enhancement of the radiation response when *cis*-DDP and irradiation were given at short time intervals (Douple & Richmond, 1982; Overgaard *et al.*, 1981; Kyriazis *et al.*, 1983; Höglmeier *et al.*, 1985; Lelieveld *et al.*, 1985).

An increasing number of clinical phase I-II studies has been carried out with combined treatments of radiotherapy and *cis*-DDP at varying dose schedules (Reimer *et al.*, 1981; Keizer *et al.*, 1984; Dewit *et al.*, 1985; Pinedo *et al.*, 1983). However, these data do not allow any firm conclusions as to the effect of *cis*-DDP in combination with radiotherapy on human normal tissues.

Attention has been drawn to increased acute normal tissue reactions due to the combined use of cancer chemotherapeutic agents with radiotherapy (Phillips *et al.*, 1976; Peckham *et al.*, 1981). Since the normal tissue tolerance is the limiting factor in the treatment of most malignancies, qualitative and quantitative knowledge of the possible interactions between *cis*-DDP and radiotherapy on normal tissues is necessary. In addition, information on the optimal dose and timing of *cis*-DDP in relation to irradiation is needed.

The present study reports on the effect of *cis*-DDP on the radiosensitivity as well as on repair of sublethal radiation damage and repopulation during fractionated radiotherapy in the lip mucosa of mice. This tissue was selected as a model for fast proliferating tissues (Ang *et al.*, 1985).

### Methods and materials

The experiments were carried out using 8 to 9 weeks old female Naval Medical Research Institute (NMR1) mice. These outbred mice were bred in specific pathogen free conditions and were transported to a conventional housing facility for treatment and follow-up. They were housed 6 per cage with free access to food and water.

Irradiations were delivered with a $^{60}Co$ gammaray unit. The dose rate at a focus-to-skin distance of 60 cm was $170\,cGy\,min^{-1}$ in some experiments and $140\,cGy\,min^{-1}$ for others. With the use of a

*Present address: MD Anderson Hospital, University of Texas System Cancer Centre and Tumor Institute, Houston, Texas, USA.

Correspondence: W. Landuyt.

Received 20 February 1986; and in revised form 10 June 1986.

© The Macmillan Press Ltd., 1986

semiclosed Ethrane® (Abbott, Belgium) anaesthesia system (Ang et al., 1982), 24 mice placed in a supine position could be treated simultaneously. The snouts of the mice were irradiated, while the remaining part of the body was shielded with 7 cm thick MCP alloy (Mining Chemical Product; m.p. 70°C) (Xu et al., 1984).

The solutions of *cis*-DDP (Platinol®; kindly provided by 'Bristol, Belgium') were prepared with sterile 0.9% saline shortly before i.p. injections. The drug was always administered in a volume of 0.25 ml per mouse. Drug toxicity was assessed using escalating doses to groups of at least 8 mice, using weight loss and lethality as parameters. In the combined modality treatments several doses of *cis*-DDP were tested. These drug doses, ranging from 6 to 13 mg kg$^{-1}$, did not result in any macroscopic change of the mouse lip mucosa. In order to exclude diurnal variations in toxicity, the experiments were always carried out between 4 and 6 p.m.

Experiments with irradiation alone were done in parallel with every combined treatment regimen. To assess the reproducibility, experiments were always repeated once or twice, with an interexperimental variability of about 5% at the isoeffect level (mean peak reaction). Acute lip mucosal reactions were scored 5-6 times per week by at least two observers during the period of 17 to 21 days following the initial irradiation treatment using a semi-quantitative scale (Xu et al., 1984). The mean peak reaction level (mean score for a group of mice) for each radiation dose point was used for constructing dose-response curves. Since *cis*-DDP administration altered the extent of oedema, the influence of this factor on the lip mucosal score was estimated by separately assessing the reaction including or excluding oedema from the calculations of average responses on several occasions. For the various treatment schedules, comparison between the reactions after irradiation alone and after irradiation combined with *cis*-DDP was also done based on the percentage of animals showing focal desquamation of the mouse lip mucosa. When the animals showed exudative reactions and/or crusting covering more than half of the lips, they were killed. During the period of scoring, mice were also weighed daily in order to assess the toxicity of the combined treatment. The mice were killed when the critical level of 35% weight loss was reached because previous experiments had demonstrated that in such circumstances lethality always followed.

### Results

### *Toxicity of* cis-*DDP alone and combined with irradiation*

A series of experiments were carried out to assess toxicity of *cis*-DDP in the female NMRI mice following single and repeated i.p. injections. For single injection the drug dose needed to induce 50% lethality within a period of 30 days (LD50/30) was found to be 21 mg kg$^{-1}$ in one experiment but later however, the LD50/30 was only 15 to 16 mg kg$^{-1}$. When 5 i.p. injections of *cis*-DDP were given at 24 h-intervals, the LD50/30 was estimated to be ~21 mg kg$^{-1}$ total dose. The latter result was obtained at the same time period during which the first single injection study was carried out. In both schedules of drug administration, the maximum *cis*-DDP dose causing no lethality was 13–14 mg kg$^{-1}$.

During the 30 days of observation, the body weight of all animals was recorded daily. The results showed no weight loss for a single injection of 6 mg kg$^{-1}$ drug and ~12% loss of body weight following a single treatment of 8 mg kg$^{-1}$ *cis*-DDP. Weight loss after 10 and 13 mg kg$^{-1}$ single injections varied from 10 to 25% with relatively large individual variations between the mice. Slightly smaller changes in body weight were observed with the repeated daily injections when comparing the same total drug doses up to 10 mg kg$^{-1}$. Following the decrease in body weight, the surviving mice however recovered to more than the initial weight within the observation period. Acute morbidity occurred between 5 and 9 days after drug injection and was always preceded by severe dehydration and dramatic loss of body weight. Macroscopically, the intestines were often swollen with a watery content. Histological preparations of the small intestine, resected immediately post-mortem, showed atrophied crypts and shortened villi.

Combining *cis*-DDP with irradiation, at any of the doses used, led in a number of experiments to a more pronounced loss of body weight than expected from the sum of weight loss of both agents alone (Table I). The decrease was observed at the time period of the maximal lip mucosal reaction and persisted for several days. The phenomenon of a greater reduction in body weight was more obvious in fractionated compared with single treatments.

### *Combined* cis-*DDP and irradiation: lip mucosal reactions*

Figure 1 shows the results of two time-line experiments combining 8 mg kg$^{-1}$ (A) or 13 mg kg$^{-1}$ (B) single i.p. *cis*-DDP injections with a single radiation dose of 15 Gy.

The drug administered at either 24 h, 6 h, 2 h, 15 min before or 15 min, 2 h, 6 h, 24 h, 72 h after the irradiation.

No enhancement of the lip mucosal reactions was recorded at any of the time-intervals used. In fact,

**Table I** Maximum weight loss for various treatment modalities.

| *Irradiation* | | | |
|---|---|---|---|
| *Scheme* | *Total dose (Gy)* | *cis-DDP* ($mg\,kg^{-1}$) | *%Max. weight loss* (±1 *s.e.*) |
| | | 1×8 | 12.2±2.1 |
| | | 1×13 | 19±2 |
| | | 5×1.2 | 0 |
| | | 5×1.6 | 5±2 |
| | | 5×2.0 | 2.7±1.3 |
| 1F | 14.5 | | 7.8±2.5 |
| 1F | 14.5 | 1×8 (2 h prior RT) | 21.8±2.6 |
| 1F | 14.5 | 1×13 (2 h prior) | 29.8±3.7 |
| 5 daily F | 28 | | 10.5±2.9 |
| 5 daily F | 28 | 5×1.2 (30 min prior RT) | 12±2.7 |
| 5 daily F | 28 | 5×1.6 (30 min prior RT) | 25.3±4 |
| 5 daily F | 28 | 5×2 (30 min prior RT) | >35[a] |

[a]Sacrificed after body weight measurement.

**Figure 1** Time-line experiments using a single i.p. injection of *cis*-DDP given at various time intervals before and after a single radiation treatment. (A) 8 $mg\,kg^{-1}$ drug combined with 15 Gy (dose rate of 140 $cGy\,min^{-1}$). (B) 13 $mg\,kg^{-1}$ drug combined with 15 Gy (dose rate of 170 $cGy\,min^{-1}$). The hatched area corresponds with the mean peak mucosal reaction (±1s.e.) following irradiation alone. Vertical bars, in this and all other figures, represent ±1s.e.

Figure 1 shows that slightly less lip mucosal reactions occurred after the concomitant use of *cis*-DDP and irradiation. However, this does not necessarily implicate a protective effect and most probably was the result of the often occurring dehydration of the *cis*-DDP treated animals leading to a reduced oedema score. This would obviously cause an underestimate of the total score relating to the acute lip mucosal reactions; the erythema and desquamation scores were not significantly altered.

In Figure 2 (A and B), the dose-response curves obtained after single irradiation alone and after i.p. injection of 13 $mg\,kg^{-1}$ *cis*-DDP 2 h prior to single irradiation doses, confirm the above mentioned findings. The reduction in the reaction score following combined treatment (Figure 2A) is not significant when the scores for oedema are excluded (Figure 2B). Also the percentage of animals showing focal desquamation of the mouse lip mucosa were similar for both treatment modalities (Table II).

Results of the investigation of a possible influence of *cis*-DDP on the extent of repair of sublethal radiation damage are presented in Figure 3. Fractionated, daily treatment of irradiation and *cis*-DDP (1.2 or 1.6 $mg\,kg^{-1}$ given 30 min before each irradiation) for 5 consecutive days resulted in less radiation damage compared with irradiation alone (Figure 3A), even when the oedema scores were not used for calculating the mean peak reactions (Figure 3B). Reduced reactions were not only observed at the day of peak reactions but were found over the whole reaction course after the combined treatment. The lower incidence of spotted desquamation in the mouse lip following the concomitant use of *cis*-DDP and irradiation is demonstrated in Table II.

Experiments combining *cis*-DDP and two radiation doses given with increasing fractionation intervals (1 to 4 h) were done to assess the possible influence of *cis*-DDP on the rate of repair of sublethal radiation damage. The results did not

**Figure 2** Dose-response curves representing the mean peak mucosal reactions as a function of administered radiation dose. *Cis*-DDP (13 mg kg$^{-1}$) is injected i.p. at 2 h prior a range of single radiation doses. Calculations were accomplished including the oedema score (A) or without the oedema score (B). ○: irradiation alone, ●: combined treatment.

**Table II** Percentages of animals showing focal desquamation of the mouse lip mucosa after irradiation alone or after irradiation combined with i.p. *cis*-DDP administration.

| *Single dose irradiations* | | | *5 daily fraction irradiations* | | | |
|---|---|---|---|---|---|---|
| *Dose (Gy)* | *% Incidence (without cis-DDP)* | *% Incidence (with cis-DDP)*[a] *13 mg kg*$^{-1}$ | *Total dose (Gy)* | *% Incidence (without cis-DDP)* | *% Incidence (with cis-DDP)*[b] *1.2 mg kg*$^{-1}$ | *1.6 mg kg*$^{-1}$ |
| 14.5 | 0 | 0 | 28.0 | 0 | 0 | 0 |
| 16.0 | 33 | 25 | 29.5 | 0 | 0 | 0 |
| 17.5 | 83.5 | 100 | 31.0 | 50 | 0 | 0 |
| 19.0 | 100 | 100 | 32.5 | 67 | 33 | 0 |

[a]Drug administered 2 h prior to irradiation; [b]each drug administration 30 min prior to each irradiation.

indicate any influence of the drug on the repair kinetics (data not shown) when the drug was injected 30 min prior to the first radiation treatment.

Figure 4 presents data obtained following the use of 2 equal sized radiation doses separated by 10 days. It was previously shown that this time interval allowed for a large amount of tissue regeneration (Ang *et al.*, 1985). In order to assess the effect of *cis*-DDP on repopulation, 5 daily drug injections were given during the interval between both fractions using either 1.2 mg kg$^{-1}$ or 1.6 mg kg$^{-1}$ *cis*-DDP to total doses of 6 mg kg$^{-1}$ or 8 mg kg$^{-1}$ respectively. The injections were given daily starting at day 3 after the first irradiation. The reaction curves (total radiation dose of 19 Gy) shown in this figure demonstrate clearly that the addition of repeated drug injections did not alter

**Figure 3** The response of the mouse lip mucosa to 5 equal radiation doses separated by 24 h without *cis*-DDP (○) and with 5 daily i.p. injections of either 1.2 mg kg$^{-1}$ (●) or 1.6 mg kg$^{-1}$ (■) drug at 30 min interval. The dose response curves of part A are constructed including the oedema scores, while in part B the oedema scores are omitted.

**Figure 4** Course of mouse lip mucosal reactions following 2 equal radiation doses of 14.5 Gy each at 10 days interval. The average reaction includes the oedema score and is plotted as a function of time after the first radiation treatment. The various schedules are: Irradiation alone (○); Irradiation with 1.2 mg kg$^{-1}$ (▲) or 1.6 mg kg$^{-1}$ (■) *cis*-DDP given daily i.p. from 3 to 7 days after the first radiation exposure.

the course of radiation responses. Similar results were also obtained for the lower radiation doses used.

### Discussion

### *Toxicity*

Our histological findings are in agreement with the marked intestinal cytotoxicity described earlier in rats and mice (Choie *et al.*, 1981; Kovacs *et al.*, 1982; Luk *et al.*, 1979; Schaeppi *et al.*, 1973). Obviously, the rapidly developing acute renal damage with *cis*-DDP doses of more than $6\,mg\,kg^{-1}$ (Stewart *et al.*, 1986) will add to the observed body weight loss and eventual lethality.

The results from our *cis*-DDP toxicity studies with the NMRI mice showed $LD_{50/30}$ variations for single i.p. injections. It was also noticed that the responses to the drug were subject to individual variations within one dose group. This is true for every group of mice receiving the same amount of *cis*-DDP. Investigations on kidney function in mice after *cis*-DDP administration showed a similarly broad range of individual acute responses at doses above $6\,mg\,kg^{-1}$ (Stewart *et al.*, 1986).

When the drug was combined with irradiation a more pronounced body weight loss occurred, also at the lowest radiation and drug doses used. This increased toxicity following the combined irradiation and *cis*-DDP treatment, as compared to the sum of both agents alone, was also reported by others (F. Stewart & L. Dewit, personal communication) and was found previously with other drugs, i.e. actinomycin D (Landuyt *et al.*, 1985) and methotrexate (von der Maase, 1984). This is probably the result of a spatial combination of normal tissue toxicity, an assumption based on the fact that evidence for systemic toxicity and lip mucosal injury showed a similar onset in time.

### *Combined* cis-*DDP and irradiation: lip mucosal reactions*

The results of our time-line experiments, in which a single injection of *cis*-DDP was given at various time intervals from 1 day before to 3 days after a single radiation exposure, showed no clear modifications of the radiation induced lip mucosal damage. Neither was there any evidence of modified radiation damage when a fixed amount of drug was delivered before or after a range of radiation doses. It was also demonstrated that the reduced scores, due to the administration of *cis*-DDP and occurring in some combined schedules, were probably the result of dehydration of the animals. Since this phenomenon leads to a reduced swelling of the lips, omission of the oedema score shows that only minor differences existed between both combined treatment and radiotherapy alone. The incidence of spotted desquamation in the mouse lip after radiation treatment was not modified with the use of *cis*-DDP. These results therefore illustrate that the radiosensitivity of this rapidly proliferating tissue does not seem to be affected by *cis*-DDP for both the time intervals and sequences used.

In agreement with this finding, absence of interaction between radiation and *cis*-DDP on the skin of mouse foot has been previously reported (Overgaard *et al.*, 1981; Lelieveld *et al.*, 1985). Some investigators, however, did show a moderate increase of skin reactions in mouse foot (von der Maase, 1984*b*) as well as in the rat foot (Douple *et al.*, 1979). A phase I/II clinical study combining radiotherapy and daily low dose *cis*-DDP for treatment of locally advanced cancers showed no clear increase of the oral mucosal reactions or skin lesions (Keizer *et al.*, 1984). Other clinical data also indicated no change of radiation induced acute skin reactions when *cis*-DDP was given concurrently with radiotherapy for treatment of solid tumours (Dewit *et al.*, 1985*a*; Reimer *et al.*, 1981). In contrast, a number of studies showed enhancement of radiation damage in the intestinal mucosa of mice when *cis*-DDP was given i.p. at various time periods before or after single radiation exposure (Luk *et al.*, 1979); von der Maase, 1984*a*). However, the effect only occurred when the drug dose exceeded $6\,mg\,kg^{-1}$. An enhancement of acute radiation damage to the small intestine in patients has also been shown to be *cis*-DDP dose dependent (Dewit *et al.*, 1985*a*).

No interaction of *cis*-DDP with the extent nor the kinetics of repair of sublethal radiation damage was demonstrated in the present study. It was actually found that, following a combined treatment of 5 daily *cis*-DDP injections given 30 min before 5 daily irradiations, the lip mucosal reactions were consistently lower compared with those after radiotherapy alone. This small but significant protective effect could not be explained by dehydration since exclusion of the oedema scores from the calculation of mean peak level of mucosal reaction did not eliminate this phenomenon. These effects may be explained partly by synchronisation occurring during the treatment course. These results on mouse lip mucosa are in agreement with the data of Bartelink *et al.* (1983) and Lelieveld *et al.* (1985) on mouse skin, demonstrating no modification of the capability to repair sublethal radiation damage. While inhibition of repair of sublethal radiation damage by *cis*-DDP has been suggested in several investigations on mouse intestinal epithelium (Dewit *et al.*, 1985*b*, Bartelink *et al.*, 1983; Burholt *et al.*, 1979), no such effect was measured when low dose rate irradiation was combined with continuous i.p. infusion of *cis*-DDP using the same biological model (Fu *et al.*, 1984).

Finally, the possible interference of *cis*-DDP with repopulation during fractionated irradiation was also investigated. Our data do not show any influence of fractionated drug treatments (6 or $8\,mg\,kg^{-1}$ total dose) on the repopulation capacity of mouse lip mucosa. Figure 4 illustrates the results obtained with a total radiation dose of 29 Gy, but similar results were obtained for lower radiation doses. Higher *cis*-DDP doses could not be tested, since in this combined treatment they always resulted in very severe toxicity and subsequent lethality.

In conclusion, these data on lip mucosa do not show an increase of the radiosensitivity nor an inhibition of repair at the cellular or tissue level. On the contrary, they indicate a minor protection (6–9%) dependent on the fractionation schedule used. Thus it follows from our results and from data in the literature that the effect of *cis*-DDP on radiation treatment is strongly dependent on the type of tissue involved. It also seems clear that large combined effects are apparent in intestinal mucosa when high drug doses are used. This could be related to the fact that at these doses of *cis*-DDP a marked independent cell killing in gut mucosa occurs from the drug alone.

There certainly remains a need for more data derived from the combination of fractionated drug and radiation treatments used concomitantly as well as alternately in a variety of normal and tumour tissues, to define the optimal use of combined modalities in clinical practice.

The present investigation was supported financially by the Algemene Spaar- en Lijfrentekas (A.S.L.K.). The authors wish to thank Bristol Laboratories, Brussels, for kindly providing the *cis*-DDP.

Thanks also to Drs H.J. Keizer, F. Stewart, A. Begg and L. Dewit for helpful suggestions and discussions, A. Camps and M. Ramaekers for technical assistance and M.-L. Detienne and E. Boyen for skillful secretarial assistance.

### References

ANG, K.K., VAN DER KOGEL, A.J. & VAN DER SCHUEREN, E. (1982). Inhalation anesthesia in experimental radiotherapy: a reliable and time saving system for multifractionation studies in a clinical department. *Int. J. Radiat. Oncol. Biol. Phys.*, **8**, 145.

ANG, K.K., XU, F.X., VANUYTSEL, L. & VAN DER SCHUEREN, E. (1985). Repopulation kinetics in irradiated mouse lip mucosa: The relative importance of treatment protraction and time distribution of irradiations. *Radiat. Res.*, **101**, 162.

BARTELINK, H. & KALLMAN, R.F. (1983). The effects of cisplatin and irradiation on tumor, skin and gut in mice. Proceedings of 25th Annual ASTR Meeting. *Int. J. Radiat. Oncol. Biol. Phys.*, **9**, 119.

BURHOLT, D.R., SCHENKEN, L.L., KOVACS, Ch.J. & HAGEMANN, R.F. (1979). Response of the murine gastrointestinal epithelium to *cis*-dichlorodiammine platinum II: Radiation combinations. *Int. J. Radiat. Oncol. Biol. Phys.*, **5**, 1377.

CHOIE, D.D., LONGNECKER, D.S. & COPLEY, M.P. (1981). Cytotoxicity of cisplatin in rat intestine. *Toxicol. Appl. Pharmacol.*, **60**, 354.

DEWIT, L., BARTELINK, H. & RÜMKE, P. (1985*a*). Concurrent *cis*-diamminedichloroplatinum (II) and radiation treatment for melanoma metastases: A pilot study. *Radiother. Oncol.*, **3**, 303.

DEWIT, L., BEGG, A.C., KÖHLER, Y., STEWART, F.A. & BARTELINK, H. (1985*b*). Influence of *cis*-diammine-dichloroplatinum (II) on mouse duodenal crypt stem cell survival after multifraction X-ray treatment. *Int. J. Radiat. Oncol. Biol. Phys.*, **11**, 1809.

DOUPLE, E.B., EATON, W.L. & TULLOH, M.E. (1979). Skin radiosensitization studies using combined *cis*-diamminedichloroplatinum II and radiation. *Int. J. Radiat. Oncol. Biol. Phys.*, **5**, 1383.

DOUPLE, E.B. & RICHMOND, R.C. (1980). In *Cisplatin: Current Status and New Developments,* Prestayko, A.W. *et al.* (eds) p. 125. Academic Press: New York.

DOUPLE, E.B. & RICHMOND, R.C. (1982). Enhancement of the potentiation of radiotherapy by platinum drugs in a mouse tumor. *Int. J. Radiat. Oncol. Biol. Phys.*, **8**, 501.

DRITSCHILO, A., PIRO, A.J. & KELMAN, A.D. (1979). The effect of *cis*-platinum on the repair of radiation damage in plateau phase Chinese hamster (V-79) cells. *Int. J. Radiat. Oncol. Biol. Phys.*, **5**, 1345.

FU, K.K., RAYNER, P.A. & LAM, K.N. (1984). Modification of the effects of continuous low dose rate irradiation by concurrent chemotherapy infusion. *Int. J. Radiat. Oncol. Biol. Phys.*, **10**, 1473.

HÖGLMEIER, F., KUMMERMEHR, J. & TROTT, K.R. (1985). Die Wirkung einer Kombinationstherapie aus Cisplatin und lokaler Bestrahlung auf ein Fibrosarkom der Maus. *Strahlenther.*, **161**, 362.

KEIZER, H.J., KAZIM, A.B.M.F., NJO, K.H. & 4 others. (1984). Feasibility study on daily administration of *cis*-diamminedichloroplatinum (II) in combination with radiotherapy. *Radiother. Oncol.*, **1**, 227.

KOVACS, C.J., BRAUNSCHWEIGER, P.G., SCHENKEN, L.L. & BURHOLT, D.R. (1982). Proliferative defects in renal and intestinal epithelium after *cis*-dichlorodiammine platinum (II). *Br. J. Cancer*, **45**, 286.

KYRIAZIS, A.P., YAGODA, A., KEREIAKES, J.G., KYRIAZIS, A.A. & WHITMORE, W.F. (1983). Experimental studies on the radiation-modifying effect of *cis*-Diamminedichloroplatinum II (DDP) in human bladder transitional cell carcinomas grown in nude mice. *Cancer*, **52**, 452.

LANDUYT, W., VAN DER SCHUEREN, E. & ANG, K.K. (1985). The effect of Actinomycin D on radiation induced reactions of the lip mucosa of mice. *Int. J. Radiat. Oncol. Biol. Phys.*, **11**, 1503.

LELIEVELD, P., SCOLES, M.A., BROWN, J.M. & KALLMAN, R.F. (1985). The effect of treatment in fractionated schedules with the combination of X-irradiation and six cytotoxic drugs on the RIF-1 tumor and normal mouse skin. *Int. J. Radiat. Oncol. Biol. Phys.*, **11**, 111.

LUK, K.H., ROSS, G.Y., PHILLIPS, T.L. & GOLDSTEIN, L.S. (1979). The interaction of radiation and *cis*-Diammine-dichloroplatinum II in intestinal crypt cells. *Int. J. Radiat. Oncol. Biol. Phys.*, **5**, 1417.

MURTHY, A.K., ROSSOF, A.H., ANDERSON, K.M. & HENDRICKSON, F.R. (1979). Cytotoxicity and influence on radiation dose response curve of *cis*-diammine-dichloroplatinum II (*cis*-DDP). *Int. J. Radiat. Oncol. Biol. Phys.*, **5**, 1411.

OVERGAARD, J. & KHAM, A.R. (1981). Selective enhancement of radiation response in a $C_3H$ mammary carcinoma by cisplatin. *Cancer Treat. Rep.*, **65**, 501.

PECKHAM, M.J. & COLLIS, C.H. (1981). Clinical objectives and normal tissue responses in combined chemotherapy and radiotherapy. *Bull. Cancer* (Paris), **68**, 132.

PINEDO, H.M., KAZIM, A.B.M.F., VAN VLIET, W.H., SNOW, G.B. & VERMORKEN, J.B. (1983). Daily *cis*-dichlorodiammineplatinum (II) as a radio-enhancer: A preliminary toxicity report. *J. Cancer Res. Clin. Oncol.*, **105**, 79.

PHILLIPS, T.L. & FU, K.K. (1976). Quantification of combined radiation therapy and chemotherapy effects on critical normal tissues. *Cancer*, **37**, 1186.

REIMER, R.R., GAHBAUER, R., BUKOWSKI, R.M. & 4 others. (1981). Simultaneous treatment with cisplatin and radiation therapy for advanced solid tumours: A pilot study. *Cancer Treat. Rep.*, **65**, 219.

RICHMOND, R.C., ZIMBRICK, J.D. & HYKES, D.L. (1977). Radiation-induced DNA damage and lethality in E.coli as modified by the antitumor agent *cis*-dichloro-diammineplatinum (II). *Radiat. Res.*, **71**, 447.

SCHAEPPI, U., HEYMAN, I.A., FLEISCHMAN, R.W. & 5 others. (1973). *Cis*-Dichlorodiammineplatinum (II) (NSC-119875): Preclinical toxicologic evaluation of intravenous injection in dogs, monkeys and mice. *Toxicol. Appl. Pharmacol.*, **25**, 230.

STEWART, F.A., BOHLKEN, S. & BARTELINK, H. (1986). Renal damage in mice after treatment with cisplatinum alone or in combination with X-irradiation. *Int. J. Radiat. Oncol. Biol. Phys.*, (In press).

VON DER MAASE, H. (1984*a*). Interactions of radiation and adriamycin, bleomycin, mitomycin C and *cis*-diamminedichloroplatinum II in intestinal crypt cells. *Br. J. Cancer*, **49**, 779.

VON DER MAASE, H. (1984*b*). Effect of cancer chemotherapeutic drugs on the radiation-induced skin reactions in mouse feet. *Br. J. Radiol.*, **57**, 697.

XU, F.X., VAN DER SCHUEREN, E. & ANG, K.K. (1984). Acute reactions of the lip mucosa of mice to fractionated irradiations. *Radiother. Oncol.*, **1**, 369.


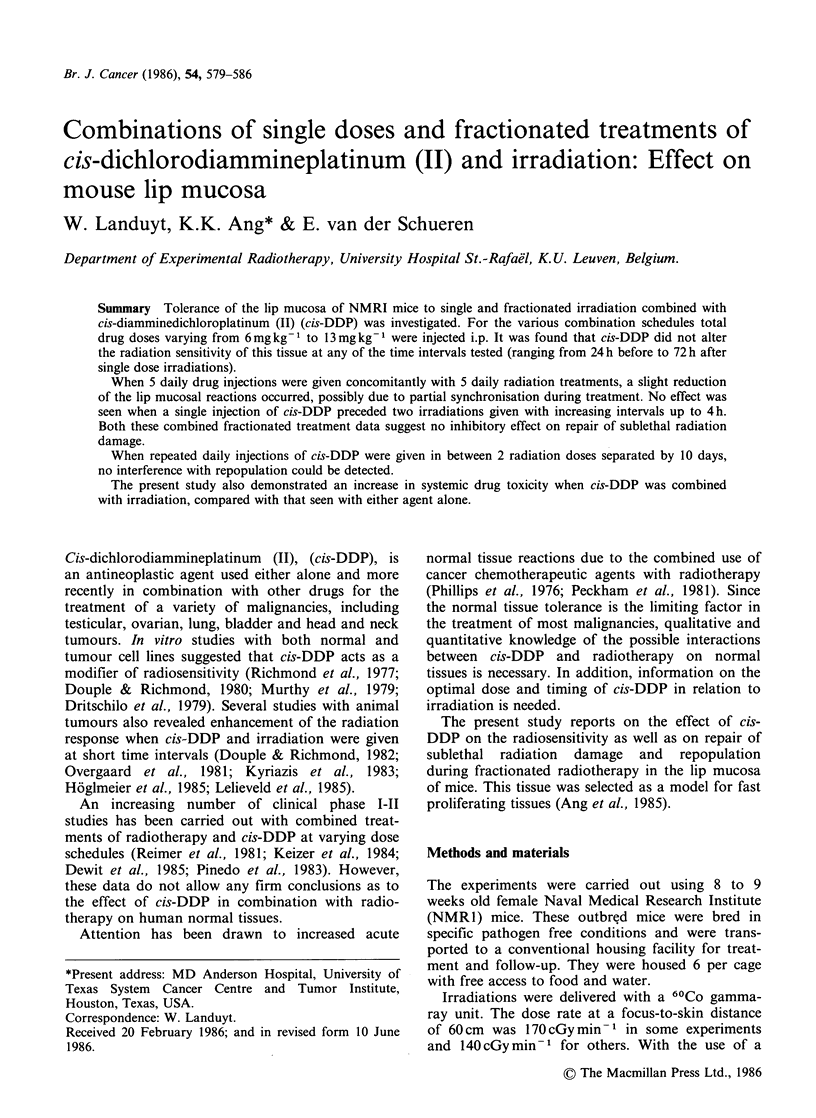

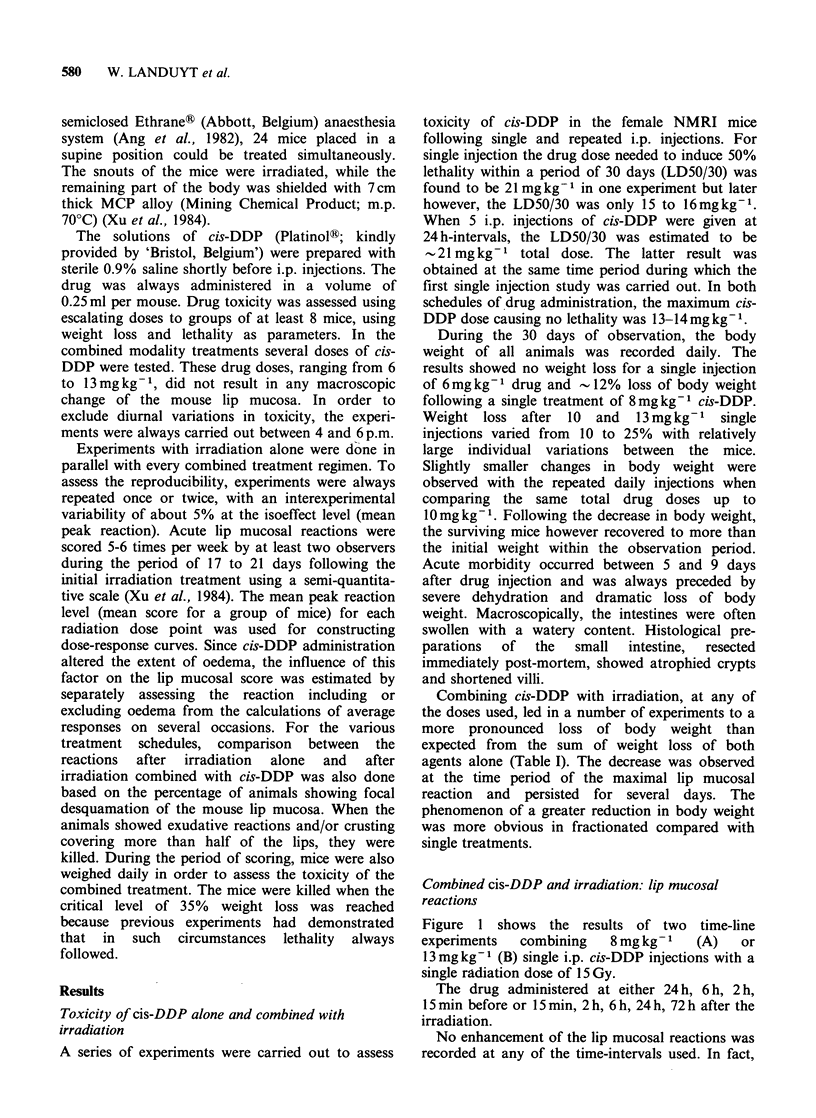

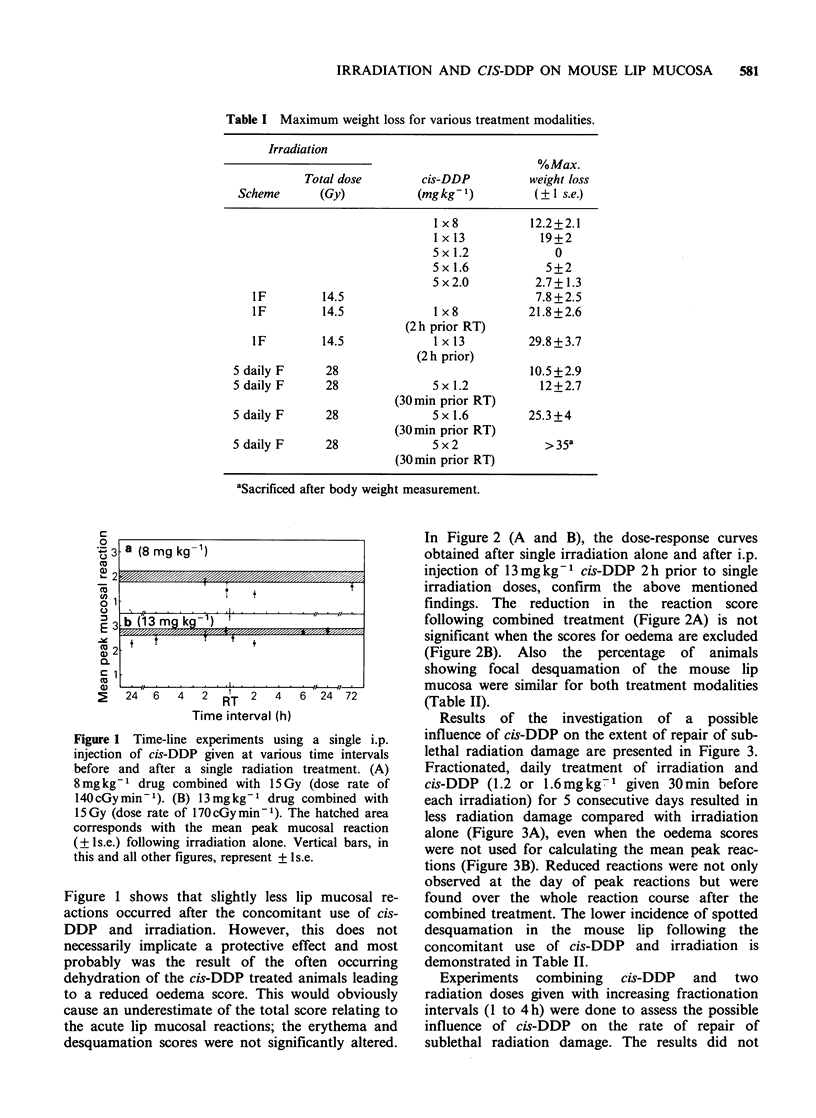

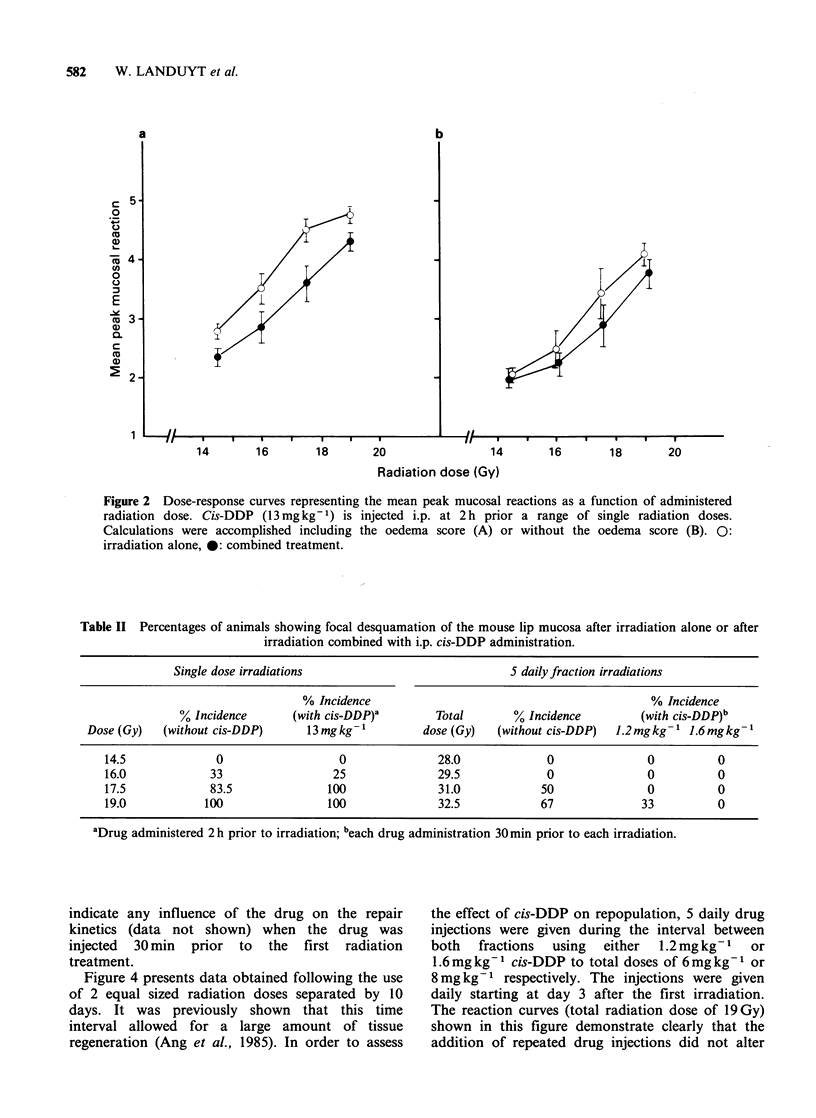

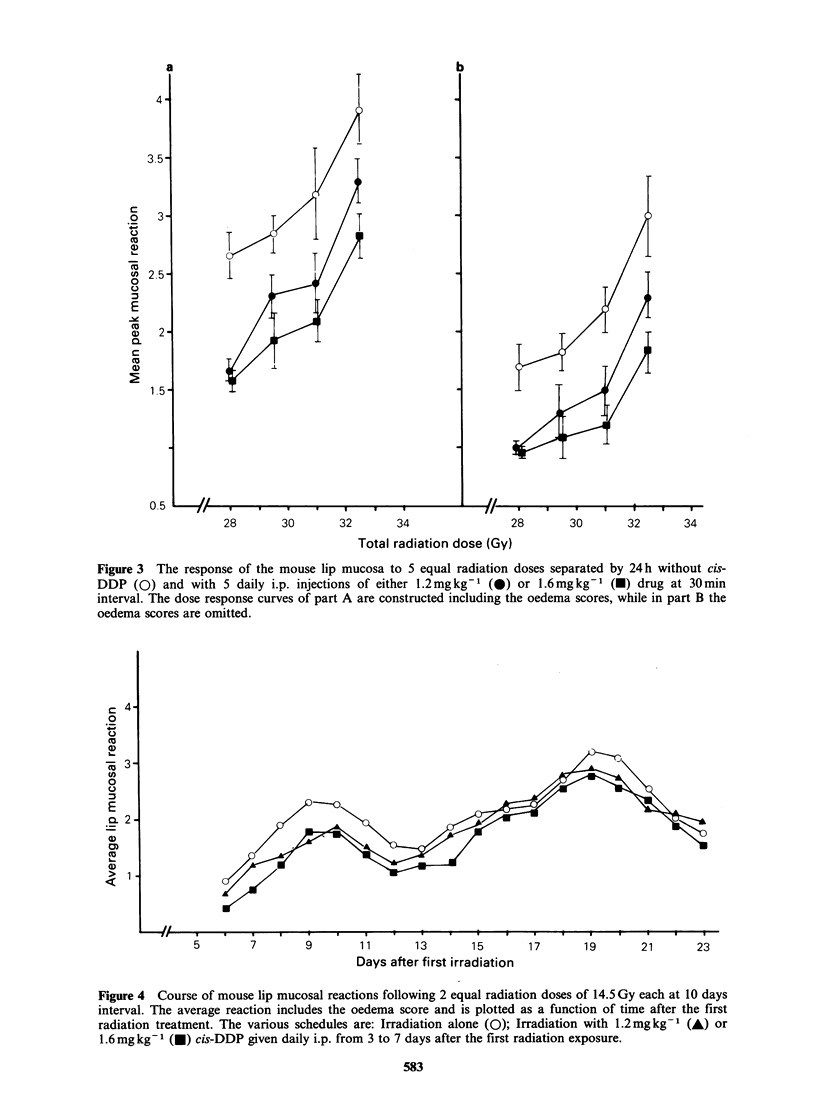

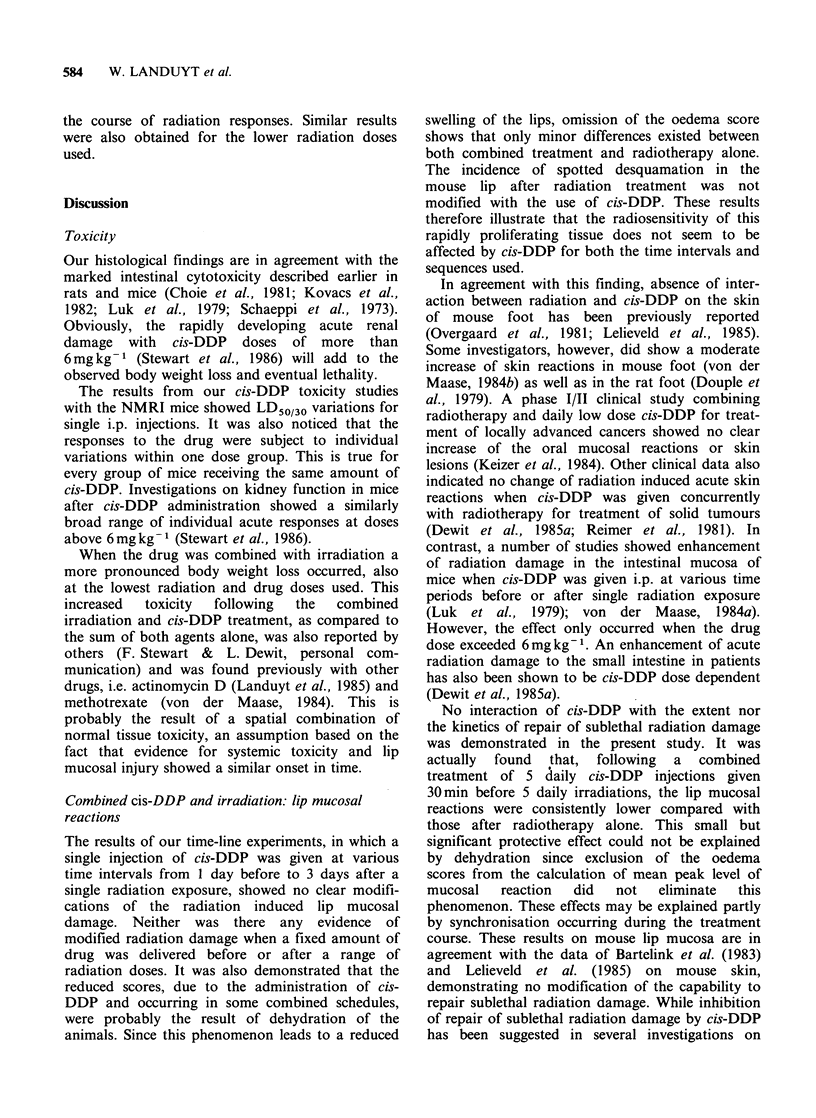

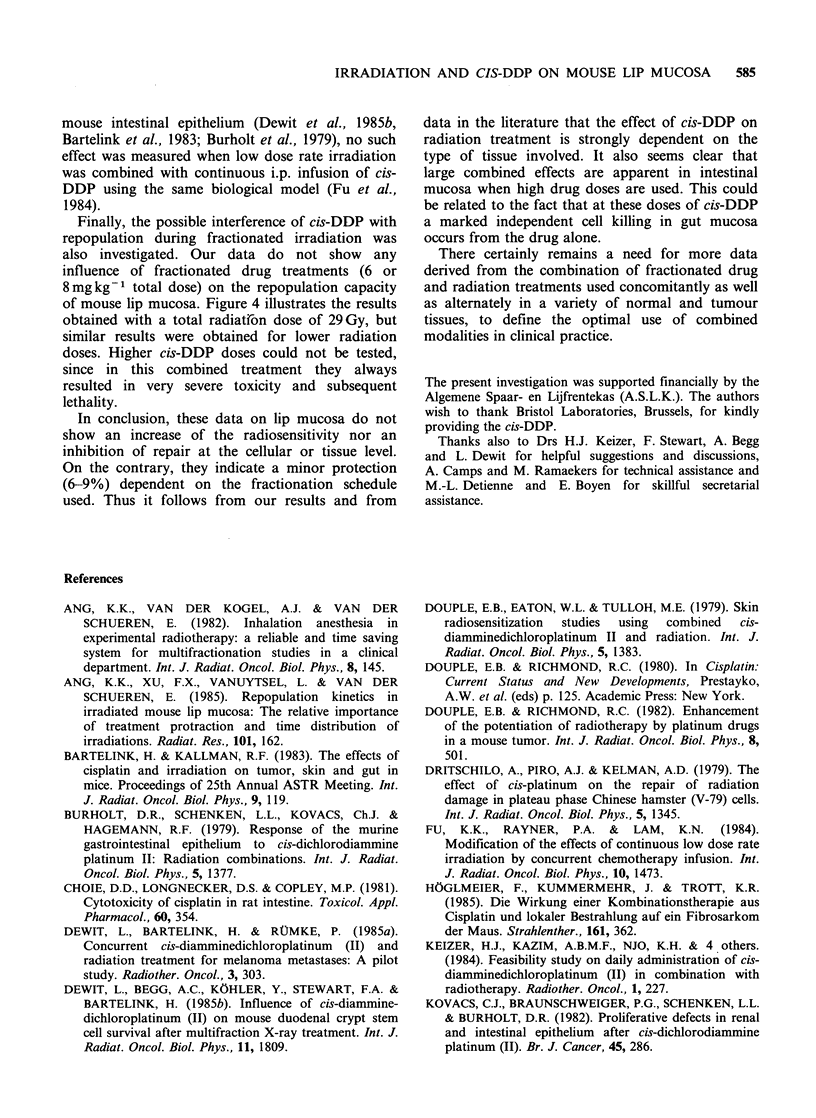

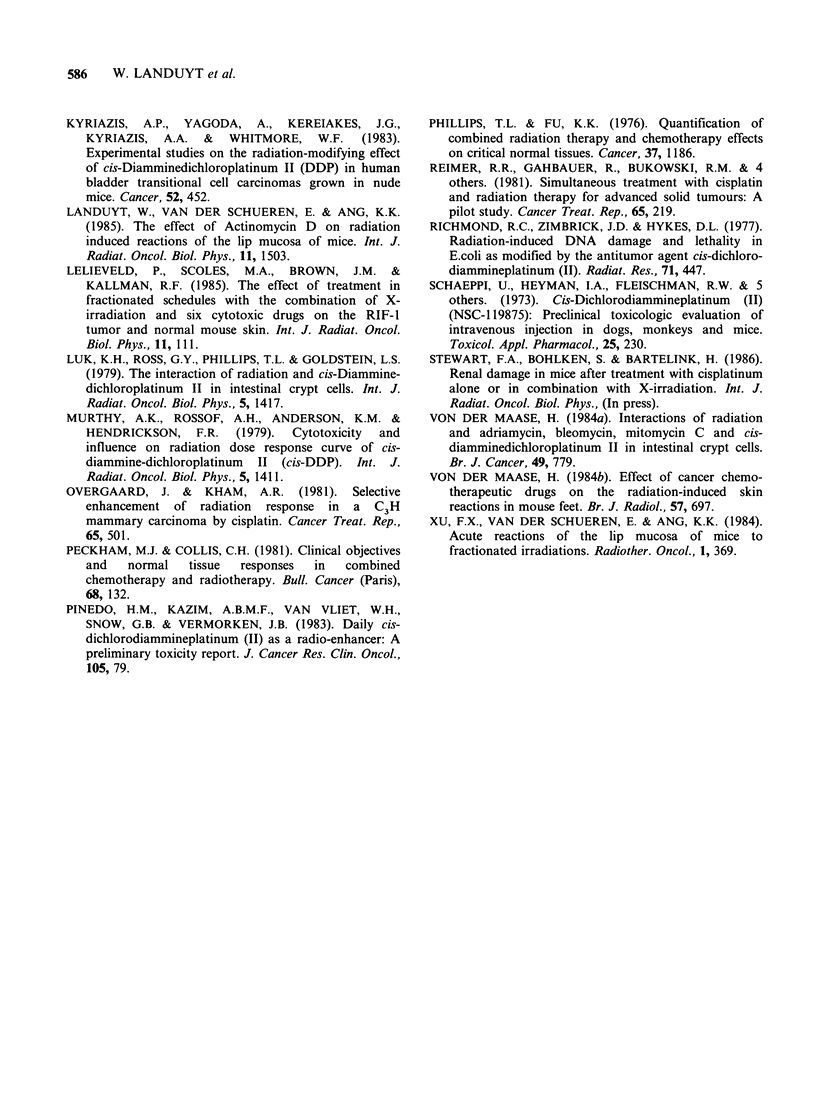


## References

[OCR_00725] Ang K. K., Xu F. X., Vanuytsel L., van der Schueren E. (1985). Repopulation kinetics in irradiated mouse lip mucosa: the relative importance of treatment protraction and time distribution of irradiations.. Radiat Res.

[OCR_00718] Ang K. K., van der Kogel A. J., van der Schueren E. (1982). Inhalation anesthesia in experimental radiotherapy: a reliable and time-saving system for multifractionation studies in a clinical department.. Int J Radiat Oncol Biol Phys.

[OCR_00740] Burholt D. R., Schenken L. L., Kovacs C. J., Hagemann R. F. (1979). Response of the murine gastrointestinal epithelium to cis-dichlorodiammineplatinum. II: radiation combinations.. Int J Radiat Oncol Biol Phys.

[OCR_00745] Choie D. D., Longnecker D. S., Copley M. P. (1981). Cytotoxicity of cisplatin in rat intestine.. Toxicol Appl Pharmacol.

[OCR_00750] Dewit L., Bartelink H., Rümke P. (1985). Concurrent cis-diamminedichloroplatinum(II) and radiation treatment for melanoma metastases: a pilot study.. Radiother Oncol.

[OCR_00756] Dewit L., Begg A. C., Köhler Y., Stewart F. A., Bartelink H. (1985). Influence of cis-diamminedichloroplatinum (II) on mouse duodenal crypt stem cell survival after multifraction X ray treatment.. Int J Radiat Oncol Biol Phys.

[OCR_00763] Douple E. B., Eaton W. L., Tulloh M. E. (1979). Skin radiosensitization studies using combined cis-dichlorodiaminneplatinum (II) and radiation.. Int J Radiat Oncol Biol Phys.

[OCR_00774] Douple E. B., Richmond R. C. (1982). Enhancement of the potentiation of radiotherapy by platinum drugs in a mouse tumor.. Int J Radiat Oncol Biol Phys.

[OCR_00780] Dritschilo A., Piro A. J., Kelman A. D. (1979). The effect of cis-platinum on the repair of radiation damage in plateau phase Chinese hamster (V-79) cells.. Int J Radiat Oncol Biol Phys.

[OCR_00786] Fu K. K., Rayner P. A., Lam K. N. (1984). Modification of the effects of continuous low dose rate irradiation by concurrent chemotherapy infusion.. Int J Radiat Oncol Biol Phys.

[OCR_00792] Höglmeier F., Kummermehr J., Trott K. R. (1985). Die Wirkung einer Kombinationstherapie aus Cisplatin und lokaler Bestrahlung auf ein Fibrosarkom der Maus.. Strahlentherapie.

[OCR_00798] Keizer H. J., Karim A. B., Njo K. H., Tierie A. H., Snow G. B., Vermorken J. B., Pinedo H. M. (1984). Feasibility study on daily administration of cis-diamminedichloroplatinum(II) in combination with radiotherapy.. Radiother Oncol.

[OCR_00732] Kopelson G., Parkinson D., Rudders R. A. (1983). Long term survivors with leptomeningeal tumor involvement.. Int J Radiat Oncol Biol Phys.

[OCR_00804] Kovacs C. J., Braunschweiger P. G., Schenken L. L., Burholt D. R. (1982). Proliferative defects in renal and intestinal epithelium after cis-dichlorodiammine platinum (II).. Br J Cancer.

[OCR_00812] Kyriazis A. P., Yagoda A., Kereiakes J. G., Kyriazis A. A., Whitmore W. F. (1983). Experimental studies on the radiation-modifying effect of cis-diamminedichloroplatinum II (DDP) in human bladder transitional cell carcinomas grown in nude mice.. Cancer.

[OCR_00820] Landuyt W., van der Schueren E., Ang K. K. (1985). The effect of actinomycin D on radiation induced reactions of the lip mucosa of mice.. Int J Radiat Oncol Biol Phys.

[OCR_00826] Lelieveld P., Scoles M. A., Brown J. M., Kallman R. F. (1985). The effect of treatment in fractionated schedules with the combination of X-irradiation and six cytotoxic drugs on the RIF-1 tumor and normal mouse skin.. Int J Radiat Oncol Biol Phys.

[OCR_00834] Luk K. H., Ross G. Y., Phillips T. L., Goldstein L. S. (1979). The interaction of radiation and cis-diamminedichloroplatinum (II) in intestinal crypt cells.. Int J Radiat Oncol Biol Phys.

[OCR_00847] Overgaard J., Khan A. R. (1981). Selective enhancement of radiation response in a C3H mammary carcinoma by cisplatin.. Cancer Treat Rep.

[OCR_00853] Peckham M. J., Collis C. H. (1981). Clinical objectives and normal tissue responses in combined chemotherapy and radiotherapy.. Bull Cancer.

[OCR_00866] Phillips T. L., Fu K. K. (1976). Quantification of combined radiation therapy and chemotherapy effects on critical normal tissues.. Cancer.

[OCR_00871] Reimer R. R., Gahbauer R., Bukowski R. M., Hewlett J. S., Groppe C. W., Weick J. K., Antunez A. R. (1981). Simultaneous treatment with cisplatin and radiation therapy for advanced solid tumors: a pilot study.. Cancer Treat Rep.

[OCR_00877] Richmond R. C., Zimbrick J. D., Hykes D. L. (1977). Radiation-induced DNA damage and lethality in E. coli as modified by the antitumor agent cis-dichlorodiammineplatinum (II).. Radiat Res.

[OCR_00883] Schaeppi U., Heyman I. A., Fleischman R. W., Rosenkrantz H., Ilievski V., Phelan R., Cooney D. A., Davis R. D. (1973). cis-Dichlorodiammineplatinum(II) (NSC-119 875): preclinical toxicologic evaluation of intravenous injection in dogs, monkeys and mice.. Toxicol Appl Pharmacol.

[OCR_00907] Xu F. X., van der Schueren E., Ang K. K. (1984). Acute reactions of the lip mucosa of mice to fractionated irradiations.. Radiother Oncol.

[OCR_00902] von der Maase H. (1984). Effect of cancer chemotherapeutic drugs on the radiation-induced skin reactions in mouse feet.. Br J Radiol.

[OCR_00896] von der Maase H. (1984). Interactions of radiation and adriamycin, bleomycin, mitomycin C or cis-diamminedichloroplatinum II in intestinal crypt cells.. Br J Cancer.

